# Whole-Brain Mapping the Direct Inputs of Dorsal and Ventral CA1 Projection Neurons

**DOI:** 10.3389/fncir.2021.643230

**Published:** 2021-04-14

**Authors:** Sijue Tao, Yihang Wang, Jundan Peng, Yang Zhao, Xiaobin He, Xuefeng Yu, Qing Liu, Sen Jin, Fuqiang Xu

**Affiliations:** ^1^Wuhan National Laboratory for Optoelectronics, Huazhong University of Science and Technology, Wuhan, China; ^2^State Key Laboratory of Magnetic Resonance and Atomic and Molecular Physics, Key Laboratory of Magnetic Resonance in Biological Systems, Wuhan Center for Magnetic Resonance, Wuhan Institute of Physics and Mathematics, Innovation Academy for Precision Measurement Science and Technology, Chinese Academy of Sciences, Wuhan, China; ^3^University of Chinese Academy of Sciences, Beijing, China; ^4^Shenzhen Key Lab of Neuropsychiatric Modulation, Guangdong Provincial Key Laboratory of Brain Connectome and Behavior, CAS Key Laboratory of Brain Connectome and Manipulation, The Brain Cognition and Brain Disease Institute (BCBDI), Shenzhen Institute of Advanced Technology, Chinese Academy of Sciences, Shenzhen, China; ^5^Materials and Interfaces Center, Shenzhen Institute of Advanced Technology, Chinese Academy of Sciences, Shenzhen, China; ^6^Shenzhen-Hong Kong Institute of Brain Science-Shenzhen Fundamental Research Institutions, Shenzhen, China; ^7^Center for Excellence in Brain Science and Intelligence Technology, Chinese Academy of Sciences, Shanghai, China

**Keywords:** dorsal CA1, ventral CA1, projection neurons, direct inputs, rabies virus tracing

## Abstract

The CA1, an important subregion of the hippocampus, is anatomically and functionally heterogeneous in the dorsal and ventral hippocampus. Here, to dissect the distinctions between the dorsal (dCA1) and ventral CA1 (vCA1) in anatomical connections, we systematically analyzed the direct inputs to dCA1 and vCA1 projection neurons (PNs) with the rabies virus-mediated retrograde trans-monosynaptic tracing system in Thy1-Cre mice. Our mapping results revealed that the input proportions and distributions of dCA1 and vCA1 PNs varied significantly. Inside the hippocampal region, dCA1 and vCA1 PNs shared the same upstream brain regions, but with distinctive distribution patterns along the rostrocaudal axis. The intrahippocampal inputs to the dCA1 and vCA1 exhibited opposite trends, decreasing and increasing gradually along the dorsoventral axis, respectively. For extrahippocampal inputs, dCA1 and vCA1 shared some monosynaptic projections from certain regions such as pallidum, striatum, hypothalamus, and thalamus. However, vCA1, not dCA1, received innervations from the subregions of olfactory areas and amygdala nuclei. Characterization of the direct input networks of dCA1 and vCA1 PNs may provide a structural basis to understand the differential functions of dCA1 and vCA1.

## Introduction

Since the dorsal and ventral hippocampi are proposed to participate in different functions (Amaral and Witter, 1989; Moser et al., 1995), evidence on the anatomical and functional segregations along the dorsoventral axis (also referred to as the longitudinal or septotemporal axis) has been cumulated (Dong et al., 2009; Fanselow and Dong, 2010; Bannerman et al., 2014; Strange et al., 2014). Generally, the dorsal hippocampus encodes spatial and cognitive information (Moser et al., 1995; Rogers and Kesner, 2006; Taube, 2007; Kim et al., 2018), while the ventral hippocampus processes emotion-related information (Kjelstrup et al., 2002; Maren and Holt, 2004; Ruediger et al., 2012; Chawla et al., 2018; McDonald et al., 2018). Structurally, spatial and non-spatial afferents to the hippocampus are relatively segregated along the dorsoventral axis (Andersen et al., 2006; Leonardo et al., 2006; Hunsaker et al., 2008; Dong et al., 2009; Fanselow and Dong, 2010; Strange et al., 2014). Differences in hippocampal connectivity along the dorsoventral axis may explain functional diversifications of the hippocampus.

As a pivotal subregion of hippocampus, the CA1 is important for the integration of different streams of information and participation in many hippocampus-related behaviors such as memory, cognition, and emotion (Lisman and Otmakhova, 2001; Cenquizca and Swanson, 2007; Igarashi et al., 2014; Kaifosh and Losonczy, 2016). Previous studies using classical tracers have provided much information about the classic extrinsic and intrinsic hippocampal circuitry of CA1. For example, CA3 projects to CA1 *via* their ipsilateral Schaffer collaterals and contralateral commissural fibers (Nakashiba et al., 2008), the entorhinal cortex (ENT) provides inputs through the temporo-ammonic pathway (Witter et al., 2000; van Strien et al., 2009), and the medial septum and diagonal band (MS-DB) areas correlate with CA1 as well (Mamad et al., 2015; Muller and Remy, 2018). Additionally, some studies have demonstrated functional and anatomical segregations between dorsal and ventral CA1 regions (referred to as dCA1 and vCA1). Literature has shown that dCA1 processes information involved in spatial location and memory (Hunsaker et al., 2008; Fanselow and Dong, 2010), while the vCA1 modulates mood-related behavior like stress and anxiety (Jacobson and Sapolsky, 1991; Parfitt et al., 2017; Jimenez et al., 2018; Padilla-Coreano et al., 2019). Distinctive heterogeneities between dCA1 and vCA1 were also found in dendritic morphology, synaptic physiology, intrinsic excitability, and gene expressions (Leonardo et al., 2006; Dougherty et al., 2012, 2013; Malik et al., 2016; Malik and Johnston, 2017; Evans and Dougherty, 2018; Dougherty, 2020).

However, traditional tracers are unable to exclusively map the cell-type specific monosynaptic input networks. There is also a relative paucity of systematic analysis and comparison of differences in the presynaptic circuit of dCA1 and vCA1 quantitatively. This paper focuses on systematic quantification and detailed analysis of the direct inputs of projection neurons (PNs) in dCA1 and vCA1. By employing the genetically modified rabies virus (RV) tracing system and Thy1-Cre transgenic mice, we represented the complex and varied circuity of CA1 along the hippocampal dorsoventral axis. Our whole-brain mapping revealed that inputs to the dCA1 and vCA1 PNs were different along the rostrocaudal axis (RC axis): vCA1 PNs directly integrated information from both intrinsic and extrinsic hippocampal subregions, while the dCA1 PNs preferentially received information from intrinsic hippocampal subregions.

## Results

### Overview of the Whole-Brain Inputs to Dorsal and Ventral CA1 Projection Neurons

**FIGURE 1:**
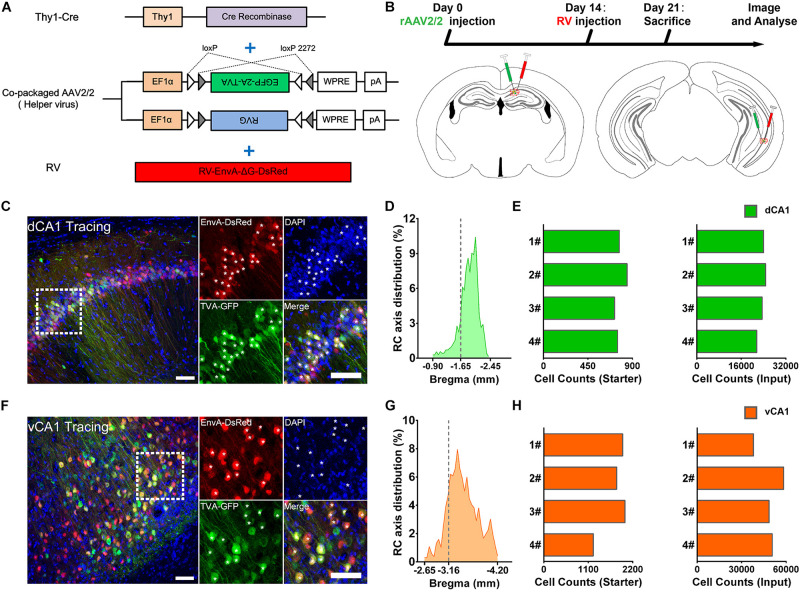
Experimental procedures for the cell-type-specific retrograde monosynaptic tracing of dorsal CA1 or ventral CA1 PNs. **(A)** Thy1-Cre transgenic mice treated with co-packaged recombinant adeno-associated virus (AAV) strains and rabies virus (RV). **(B)** Experimental design showing timeline and injection site for dCA1 and vCA1 tracing group. **(C,F)** Representative images of coronal brain sections containing the injection sites and the magnifications of the starter cells (**C**, the dorsal CA1 tracing group; **(E)**, the ventral CA1 tracing group). The starter cells coexpressing GFP and DsRed are indicated by white asterisks. Scale bar: 50 μm. **(D,G)** Distribution patterns of the starter cells in the injected CA1 regions as detected along the RC axis. **(E,H)** Numbers of the starter cells and numbers of input neurons in whole brain in each mouse of dCA1 and vCA1 tracing groups (quantified with all slices; *N* = 4).

We applied the RV-based monosynaptic tracing system to Thy1-Cre mice (*N* = 4 in each tracing group) in which the hippocampal PNs expressed Cre recombinase (Figure 1A) to identify the monosynaptic inputs of PNs in dCA1 and vCA1. For both tracing groups, the starter cells were restricted to the injected dCA1 and vCA1 areas and distributed across the RC range of the injected site with peak around the targeted coordinates (Figures 1D,G). We counted the number of the starter cells (coexpressing GFP and DsRed) and RV-labeled input neurons (only expressing DsRed) within each brain region or subregion (Figures 1C,F).

For quantitative analysis, we counted 21,526–24,727 input neurons in each brain of the dCA1 tracing group and 37,971–58,357 input neurons in each brain of the vCA1 tracing group (Figures 1E,H; see Supplementary Table 1 for specific data values). Then, we calculated the mean convergence index (defined as the number of whole-brain presynaptic DsRed+ cells divided by the number of starter cells) of both tracing groups. The mean convergence index of the dCA1 tracing group was 30.54 ± 1.60 (mean ± SEM) and that of the vCA1 tracing group was 28.40 ± 1.15. Since there was no significant difference in convergence index (*P* = 0.317) between the two experiment groups, the differences in the monosynaptic afferents represent the differences in the connection strength between the CA1 PNs and their upstream neurons.

**FIGURE 2:**
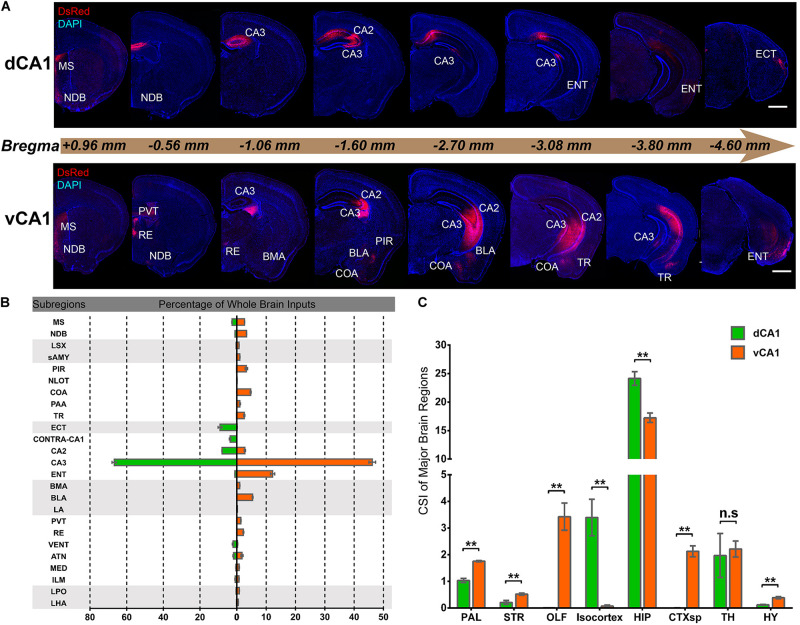
Distribution patterns of whole-brain inputs to dorsal or ventral CA1 PNs. **(A)** Representative coronal sections showing labeling of monosynaptic inputs to the dorsal or ventral CA1 PNs along the RC axis. Scale bar: 1 mm. **(B)** The proportion of input neurons within subregions of the whole brain in two tracing groups, quantified with all slices (left, dCA1 tracing group; right, vCA1 tracing group). **(C)** The CSI of eight major input brain regions, quantified with all slices. n.s., no significant difference; **P < 0.01.

Quantitative analysis of the whole-brain connections to the dCA1 and vCA1 PNs revealed that they both received extensive inputs from the brain regions along the RC axis (Figure 2A). To compare the input distribution patterns in each brain region of the two groups, the number of the input neurons within each brain region from bilateral hemispheres was normalized relative to the total number of input neurons in the whole brain. Our results showed that most of the inputs to the dCA1 PNs were observed within the HIP (79.19 ± 2.12%), followed by isocortex (10.23 ± 2.53%), and thalamus (TH) (6.10 ± 2.33%). The other regions, such as pallium (PAL) (3.42 ± 0.34%), striatum (STR) (0.28 ± 0.08%), and hypothalamus (HY) (0.40 ± 0.08%) were sparsely labeled (Figures 2A,B). Meanwhile, our results revealed that most of the input neurons to vCA1 PNs were also from the HIP (61.09 ± 1.70%), along with minor contributions of widely distributed inputs from TH (7.98 ± 1.28%), isocortex (0.26 ± 0.14%), PAL (6.25 ± 0.22%), STR (1.24 ± 0.09%), and HY (1.39 ± 0.19%). However, the olfactory area (OLF) (12.01 ± 1.41%) and amygdala nuclei of cortical subplate (CTXsp) (7.51 ± 0.53%) both projected to the vCA1 but not dCA1 PNs (Figures 2A,B).

### Both Dorsal and Ventral CA1 Projection Neurons Receive Extensive Intrahippocampal Inputs

**FIGURE 3:**
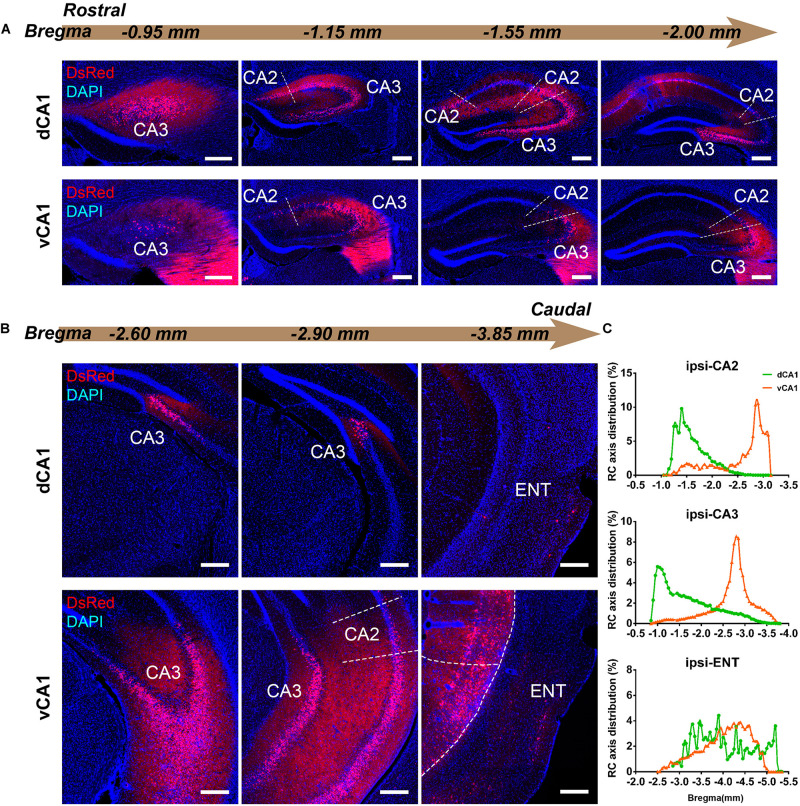
Inputs to CA1 PNs from ipsilateral HIP subregions. **(A,B)** Representative images showing distributions of the input neurons located in ipsilateral HIP. Scale bar: 250 μm. **(C)** Distribution patterns of the input neurons along the RC axis in different subregions of the ipsilateral HIP. In the ipsilateral HIP, the input neurons were found located in CA2, CA3, and ENT in both tracing groups.

**FIGURE 4:**
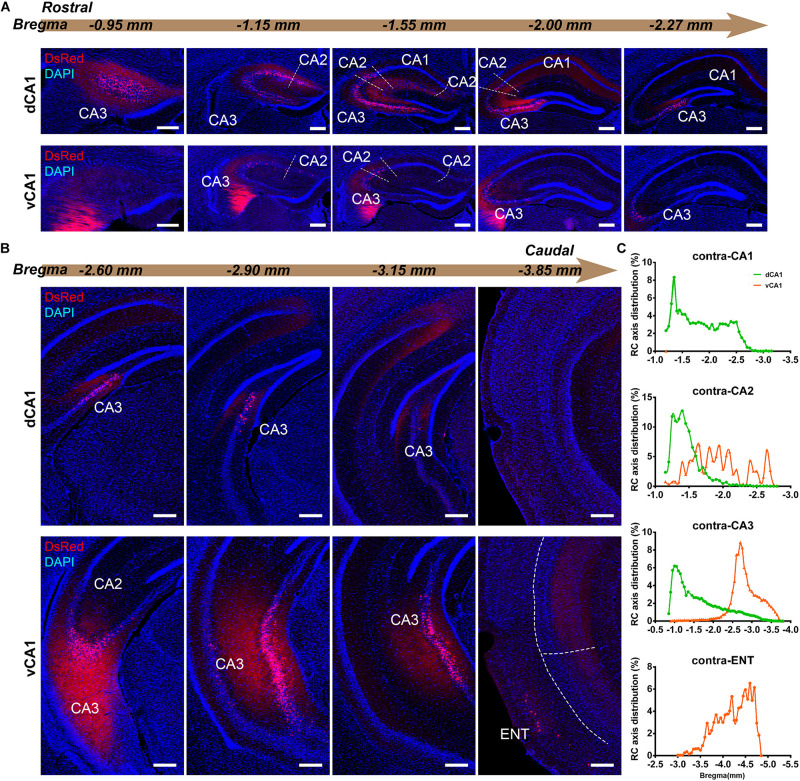
Inputs to CA1 PNs from contralateral HIP subregions. **(A,B)** Representative images showing distributions of the input neurons located in contralateral HIP. Scale bar: 250 μm. **(C)** Distribution patterns of the input neurons along the RC axis in different subregions of the contralateral HIP. In the contralateral HIP, the input neurons were found located in the CA1, CA2, and CA3 for the dorsal CA1 tracing group and in the CA1, CA2, CA3, and ENT for the ventral CA1 tracing group.

**FIGURE 5:**
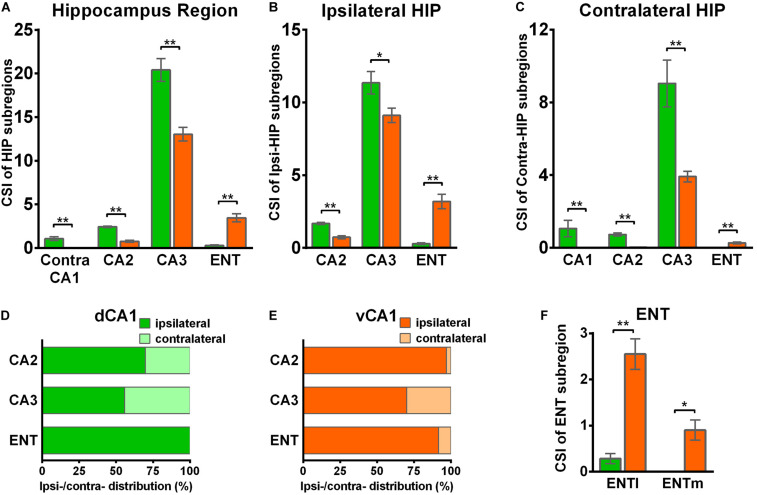
Distribution patterns of HIP inputs between the two CA1 tracing groups. **(A)** CSI of the bilateral inputs in the different HIP subregions between two CA1 tracing groups. **(B)** CSI of the inputs in the ipsilateral HIP subregions between two CA1 tracing groups. **(C)** CSI of the inputs in the contralateral HIP subregions between two CA1 tracing groups. **(D)** Distributions of ipsilateral and contralateral input neurons within five subregions of HIP. **(E)** Distributions of ipsilateral and contralateral input neurons within five subregions of HIP. **(F)** CSI of the inputs in bilateral ENT subregions. n.s., no significant difference; *P < 0.05; **P < 0.01.

We found it was very hard to separate ipsilateral CA1-to-CA1 and ipsilateral SUB-to-CA1 inputs near the injection site. Therefore, we analyzed the intrahippocampal inputs in the contralateral CA1, bilateral CA2, bilateral CA3, and bilateral ENT in this paper. Our results showed that the HIP projects to both dCA1 and vCA1 PNs (Figures 2B, 3, 4), dominantly from the ipsilateral intrahippocampus in both tracing groups (Figures 5D,E and Supplementary Table 2). The quantitative comparison showed that the bilateral intrahippocampal monosynaptic inputs to dCA1 PNs were stronger than those to vCA1 PNs (dCA1: 24.15 ± 1.19; vCA1: 17.24 ± 0.83, *P* < 0.01) (Figure 2C). Meanwhile, we found that the intrahippocampal inputs to dCA1 and vCA1 PNs shared quite different distribution patterns along both transverse and rostrocaudal axes (Figures 3, 4).

CA3 projects densely to the CA1 region *via* the Schaffer Collateral pathway and contralateral commissural pathway (Andersen et al., 2006; Schultz and Engelhardt, 2014). According to our calculations, CA3 inputs were made up of 84.29 ± 1.52% and 75.50 ± 2.56% of the total HIP inputs to the dCA1 and vCA1 PNs, respectively, as the largest input contribution to both tracing groups. Between the two groups, we found that the connection strength of CA3-dCA1 PNs and CA3-vCA1 PNs differed quantitatively (dCA1: 20.40 ± 1.29 versus vCA1: 13.03 ± 0.78, *P* < 0.01) (Figure 5A). The input strength differences of CA3 to dCA1 and vCA1 were bilateral and significant in contralateral (dCA1: ipsi-CSI = 11.36 ± 0.77, vCA1: ipsi-CSI = 9.11 ± 0.49, *P* < 0.05; dCA1: contra-CSI = 9.04 ± 0.64; vCA1: contra-CSI = 3.91 ± 0.30, *P* < 0.01) (Figures 5B,C). Along the radial axis, the CA3 input neurons of both tracing groups were mostly located in both the sublayer and deep layer of the stratum pyramidale (SP) (Figures 3A,B). However, both the dorsoventral and transverse distributions of the CA3 input cells were quite different between the two groups. The bilateral CA3 input neurons of the dCA1 group mostly resided in the rostral part (Figures 3, 4). The bilateral CA3 input neurons of the vCA1 group disseminated in both rostral and caudal parts but were largely located in the caudal part (Figures 3C, 4C). For the dCA1 group, the input neurons in the rostral part of bilateral CA3 were distributed in both distal and proximal regions (Figures 3A, 4A), and the input neurons in the caudal part of CA3 were only found in the proximal region bilaterally (Figures 3B, 4B). For the vCA1 group, input neurons in the rostral part of CA3 were mostly situated in the distal region bilaterally (Figures 3A, 4A), and the input neurons in the caudal part of CA3 were located in the middle region bilaterally (Figures 3B, 4B).

We have found some DsRed+ neurons in contralateral CA1 of the dCA1 but not the vCA1 tracing group (Figure 4). Our analysis demonstrated that contralateral CA1 made almost no interhemispheric/contralateral connections to vCA1 PNs (only two cells were found in mouse 1 in contralateral CA1 of the vCA1 tracing group, data not shown) (Figures 4A,B). Some labeled input neurons were discovered in the contralateral CA1 for dCA1 PNs (dCA1: 1.05 ± 0.22) (Figures 4, 5C). Furthermore, we analyzed the cell body distribution details of input neurons in contra-CA1 of the dCA1 tracing group. Here, the PNs received integrated contralateral inputs from the distal region in rostral CA1 (Figure 4A), with very few projections from the caudal CA1 (Figure 4B).

The entorhinal area (ENT) is regarded as the core of the parahippocampal region, since it has extensive reciprocal connections with the hippocampal region. In our data, ENT was an essential input source of whole brain to the vCA1 but not dCA1 PNs (dCA1: 0.95 ± 0.19%; vCA1: 12.19 ± 1.46%). Analysis revealed that vCA1 PNs received more inputs from ENT than dCA1 (dCA1: 0.29 ± 0.06 versus vCA1: 3.4 ± 0.47, *P* < 0.01) (Figure 5A). Meanwhile, in the dCA1 group, the PNs only integrated inputs from ipsilateral ENT (Figure 3B). In the vCA1 group, the PNs integrated inputs from bilateral ENT (Figures 3B, 4B). Of these, the lateral ENT (ENTl) projections are stronger than the medial ENT (ENTm) in both groups (Figure 5F). In the case of ENTl projections, a significant difference was found between the two groups, with stronger connections in the ENTl-vCA1 projection than in the ENTl-dCA1 (dCA1: 0.28 ± 0.06 versus vCA1: 2.55 ± 0.33, *P* < 0.01) (Figure 5F). No ENTm projections were found in the dCA1 tracing group (Figure 5F). Furthermore, the distribution patterns of ENT input neurons were different along the RC axis. In the dCA1 tracing group, the labeled neurons were distributed evenly along the RC axis in ipsilateral ENT (Figure 3C). In the vCA1 tracing group, the number of input neurons increased along the RC axis in bilateral ENT (Figures 3C, 4C).

Earlier researches reported that the projections from the CA2 to CA1 spread along the dorsoventral axis of the hippocampus (Ropireddy et al., 2012; Kohara et al., 2014; Dudek et al., 2016). In our results, robust labeling signals were observed in CA2 in both tracing groups. The analysis showed that dCA1 appears to receive stronger CA2 inputs relative to vCA1 bilaterally, with CSIs being 2.41 ± 0.08 and 0.76 ± 0.12, respectively (*P* < 0.01) (Figure 5A). The input CSIs of ipsilateral CA2 to dCA1 and vCA1 PNs were 1.68 ± 0.08 and 0.73 ± 0.11 (*P* < 0.01) and the input CSIs of contralateral CA2 to dCA1 and vCA1 PNs were 0.73 ± 0.04 and 0.02 ± 0.01 (*P* < 0.01) (Figures 5B,C). Essentially, all of the labeled CA2 cells were located in the SP (Figures 3, 4). In both groups, the labeled neurons in CA2 were mostly found in the ipsilateral part (Figures 5D,E). For the dCA1 tracing group, the input cells in CA2 were mostly located in the rostral hippocampus (Figures 3A,C, 4A,C). For the vCA1 tracing group, the input cells in CA2 were mostly located in the caudal hippocampus (Figures 3B,C, 4B,C).

### Both Dorsal and Ventral CA1 Projection Neurons Receive Pallidum and Cortical Inputs

**FIGURE 6:**
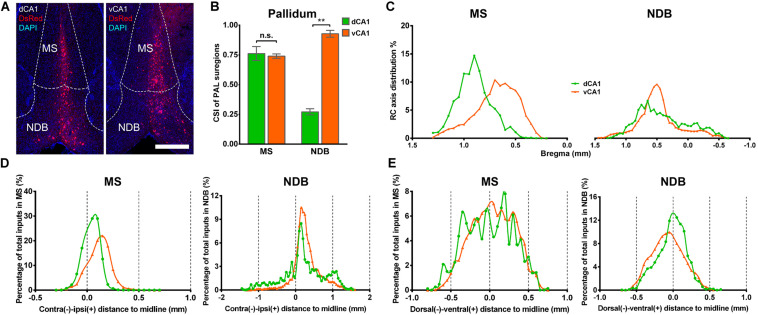
Distribution patterns of bilateral inputs in PAL subregions between the two CA1 tracing groups. **(A)** Representative images showing MS and NDB inputs to the dorsal or ventral CA1 PNs. Scale bar: 500 μm. **(B)** CSI of the inputs in bilateral PAL subregions. **(C)** Distribution patterns of the input neurons along the RC axis in the bilateral MS or NDB. **(D)** The contralateral-ipsilateral distribution patterns of monosynaptic inputs in MS or NDB of two tracing groups. **(E)** The dorsal-ventral distribution patterns of monosynaptic inputs in MS or NDB of two tracing groups. n.s., no significant difference; **P < 0.01.

In both the dCA1 and vCA1 tracing groups, the input neurons were found distributed widely in the medial septal complex subregion of PAL (Figure 6A). The inputs from the medial septal complex to the hippocampus play important roles in hippocampal spatial representation and cognition (Mamad et al., 2015; Jiang et al., 2018; Muller and Remy, 2018). As shown in Figure 6B, the inputs from MS to dCA1 and vCA1 PNs were similar (dCA1: 0.76 ± 0.06 versus vCA1: 0.74 ± 0.02, *P* = 0.748), while NDB contributed more direct afferents to vCA1 than dCA1 with CSIs of 0.93 ± 0.03 and 0.27 ± 0.03 (*P* < 0.01), respectively (Figure 6B). The input neurons in the MS/NDB region shared similar distribution patterns along the RC axis and the dorsal-ventral line between the two groups (Figures 6C,E). In both groups, the input neurons in MS/NDB were mostly located in the ipsilateral hemisphere (Figure 6D).

**FIGURE 7:**
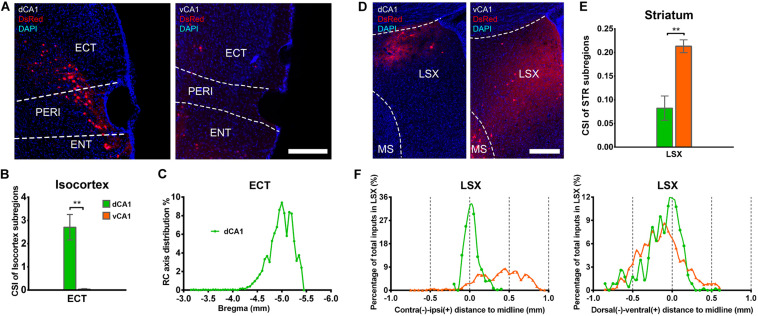
Distribution patterns of bilateral isocortex and STR inputs between the two CA1 tracing groups. **(A)** Representative images showing labeling of monosynaptic inputs to the dorsal or ventral CA1 PNs in ipsilateral ECT. Scale bar: 250 μm. **(B)** CSI of the bilateral inputs in ECT. **(C)** Distribution patterns of the bilateral input neurons to dorsal CA1 PNs along the RC axis in ECT. **(D)** Representative images showing LSX inputs to the dorsal or ventral CA1 PNs. Scale bar, 250 μm. **(E)** CSI of the inputs in bilateral STR subregions-LSX. **(F)** The contralateral-ipsilateral and dorsal-ventral distribution patterns of monosynaptic inputs in LSX of two tracing groups. **P < 0.01.

Our results showed that the notable input region in isocortex was the ipsilateral ectorhinal area (ECT) (Figure 7A), demonstrating that CA1 is the direct target of the posterior cortex. The connection strength between ECT and dCA1 was much stronger than that of vCA1, with CSIs of 2.70 ± 0.55 versus 0.04 ± 0.02 (*P* < 0.01) (Figure 7B). The density of labeled neurons in ECT increased along the RC axis in the dCA1 group (Figure 7C). As the ECT was rarely labeled in the vCA1 group (Figure 7B), the RC axis distribution was not shown. Furthermore, dCA1 received bilateral ECT inputs, while all the inputs to vCA1 were limited in ipsilateral ECT.

### Both Dorsal and Ventral CA1 Projection Neurons Receive Inputs From the Striatum, the Hypothalamus, and the Thalamus

Our data revealed that a few subregions in STR, HY, and TH yielded weak projections to both dCA1 and vCA1 PNs with CSI < 1.00. The LSX, subregion of STR, provided projections to both dCA1 and vCA1 PNs. In our data, LSX generated more inputs to vCA1 than dCA1 PNs (dCA1: 0.08 ± 0.03 versus vCA1: 0.21 ± 0.01, *P* < 0.05) (Figures 7D,E). The labeled neurons in LSX shared quite different distribution patterns along the medial-lateral line. In the dCA1 group, the percentage of input neurons has a peak in the midline, while the input neurons spread evenly in vCA1 group along the medial-lateral line. In addition, we found that the labeled neurons in LSX shared similar distribution patterns along the dorsal-ventral line for both the two groups (Figure 7F).

**FIGURE 8:**
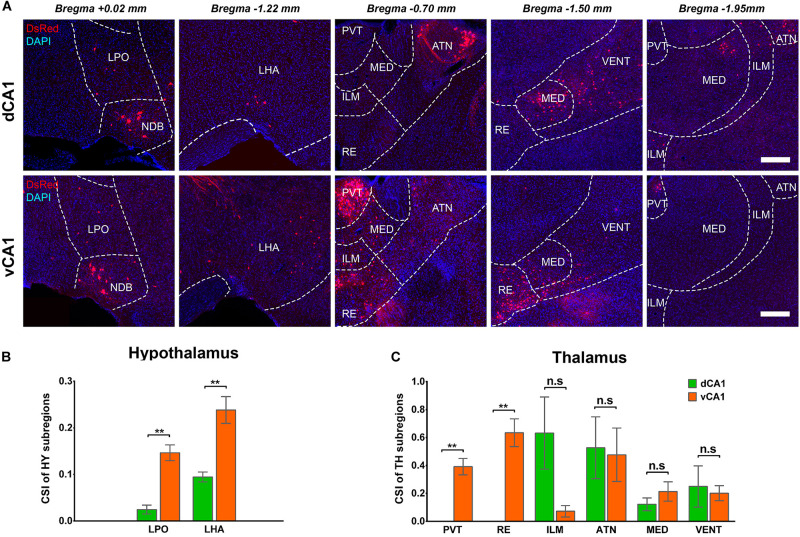
Distribution patterns of HY and TH inputs between the two CA1 tracing groups. **(A)** Representative images showing inputs from the selected subregions of HY and TH to the dCA1 or vCA1 PNs. Scale bar: 250 μm. **(B)** CSI of the inputs in HY subregions. **(C)** CSI of the inputs in TH subregions. n.s., no significant difference; **P < 0.01.

The connection strength of HY-dCA1 and HY-vCA1 differed significantly (dCA1: 0.12 ± 0.02 versus vCA1: 0.38 ± 0.04, *P* < 0.01) (Figure 2C). The discrete subregions, including LHA and LPO, projected significantly more to vCA1 than dCA1 PNs (LHA: dCA1: 0.02 ± 0.01 versus vCA1: 0.15 ± 0.02, *P* < 0.01; LPO: dCA1: 0.09 ± 0.01 versus vCA1: 0.24 ± 0.03, *P* < 0.01) but both weakly (Figures 8A,B).

A few thalamus subregions projected sparsely to both dCA1 and vCA1 PNs. Their inputs to dCA1 and vCA1 PNs were not significantly different, for ILM (CSI: 0.25 ± 0.15 versus 0.20 ± 0.05), ATN (CSI: 0.53 ± 0.22 versus 0.48 ± 0.19), MED (CSI: 0.12 ± 0.05 versus 0.21 ± 0.07), and VENT (CSI: 0.63 ± 0.26 versus 0.07 ± 0.04) (Figures 8A,C). However, two thalamus subregions, the midline nucleus reuniens (RE, CSI = 0.63 ± 0.10) and, to a lesser degree, the paraventricular nucleus of the thalamus (PVT, CSI = 0.39 ± 0.06) directly projected to vCA1, not dCA1 PNs (Figures 8A,C). Previous research found that by injecting traditional retrograde tracers in CA1, the RE form a predominant contact on both dCA1 and vCA1 (Hoover and Vertes, 2012). Our results demonstrated that vCA1 PNs received significant inputs from the RE (CSI = 0.63 ± 0.10). However, in the dCA1 group, no cells in RE were labeled in DsRed (Figures 8A,C).

### Ventral but Not Dorsal CA1 Projection Neurons Receive Inputs From Amygdala Nuclei and Olfactory Areas

**FIGURE 9:**
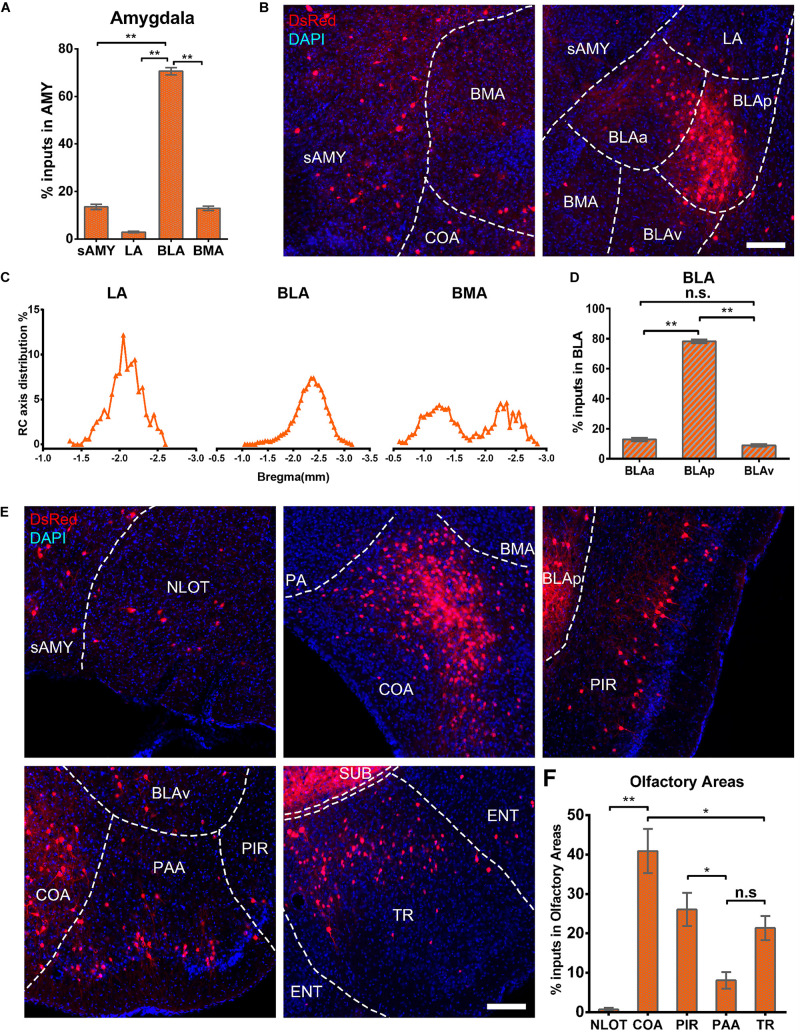
Amygdala nuclei and OLF inputs to ventral CA1. **(A)** Distribution of the input neurons within the subregions of amygdala nuclei. **(B)** Representative images showing inputs from the subregions of amygdala nuclei to the projection cells of ventral CA1. Scale bar: 250 μm. **(C)** Distribution patterns of the input neurons along the RC axis in LA, BMA, and BLA. **(D)** Distribution of the input neurons within the subregions of BLA. The BLA was mainly divided into the BLAa, BLAp, and BLAv. **(E)** Representative images showing presynaptic neurons in the subregions of OLF to the projection cells of ventral CA1. Scale bar: 250 μm. **(F)** Distribution of the input neurons within subregions of the OLF. n.s., no significant difference; *P < 0.05; **P < 0.01.

The amygdala nuclei only projected ipsilaterally to vCA1 PNs, with the basolateral nuclei of the amygdala (BLA) contributing the highest proportions of amygdala inputs [70.63 ± 3.03%, *F*(2,9) = 285.143, *P* < 0.01], followed by striatum-like amygdala groups (sAMY), basomedial amygdala nucleus (BMA), and the lateral amygdala nucleus (LA) with the percentage of 13.56 ± 2.23, 12.92 ± 1.84, and 2.89 ± 0.70%, respectively (Figures 9A,B). The RC axis distributions of the input neurons were inconsistent between the subregions of amygdala nuclei in CTXsp (Figure 9C). Within BLA, the afferent neurons were largely located in the posterior part (BLAp) with 78.23 ± 2.52% [*F*(2,9) = 1,339.696, *P* < 0.01], with fewer in the anterior (BLAa) and ventral BLA (BLAv) (12.85 ± 2.14, 8.92 ± 1.62%) (Figure 9D). The results indicated that BLAp may play a more important role in BLA-vCA1 circuit-related behavior.

Ventral CA1 PNs received significant monosynaptic inputs from OLFs, including the nucleus of the lateral olfactory tract (NLOT, 0.68 ± 0.39%), piriform area (PIR, 26.05 ± 4.24%), cortical amygdalar area (COA, 40.90 ± 5.60%), postpiriform transition area (TR, 21.35 ± 3.1%), and the piriform-amygdalar area (PAA, 8.07 ± 2.08%) [*F*(4,15) = 19.568, *P* < 0.01]. Among them, the COA, PIR, and TR were the fundamental input sources and contributed about 90% of total olfactory inputs, while NLOT and PAA were sparsely labeled (Figures 9E,F). Our results indicated that vCA1 PNs may be modulated by olfactory information.

## Discussion

In this study, our findings highlighted the heterogeneity in monosynaptic inputs of CA1 PNs across the longitudinal axis of the hippocampus. Our results corroborated with some previous tracing studies using traditional tracers, but in a cell-type-specific manner. As there are relatively few papers with quantitative analysis, we described proportions and distributions of whole-brain inputs to the dCA1 and vCA1 PNs and quantitatively compared the relative contributions of ipsilateral and contralateral projections from the whole brain. In this study, we provided a systematic and quantitative description of whole-brain direct inputs of CA1 PNs in separate dorsal and ventral parts, with statistical evaluation.

Our results showed that the dCA1 and vCA1 PNs shared some upstream brain regions/subregions but displayed distinctive input organizations of their own. The key findings of this study are that we confirmed that the intrahippocampal circuitries provide the majority of the direct afferents to PNs in both dCA1 and vCA1 (Figures 2B,C), and quantitative analysis revealed that PNs in the dCA1 received much stronger intrahippocampal monosynaptic inputs. Specifically, PNs in the dCA1 receive higher input from CA2 and CA3, while PNs in the vCA1 receive higher input from CA3 and ENT (Figure 5A). Regarding the extrahippocampal circuitry, we found some minor inputs to CA1, which was previously underexplored, such as ECT to dCA1. We also determined some inputs which only projected to vCA1 but not dCA1 PNs, such as RE, PVT, OLF, and amygdala nuclei.

### Monosynaptic Intrahippocampal Input Patterns to Different Subarea of CA1 Projection Neurons

Our results showed that most direct intrahippocampal connections of CA1 PNs were organized as a topographic gradient across the rostrocaudal hippocampus (Figures 3, 4). The input neurons of dCA1 PNs in bilateral CA2, CA3, and contralateral CA1 exhibited a decreasing gradient along the RC axis, while that of vCA1 PNs in ipsilateral CA2 and bilateral CA3 and ENT presented the increasing gradient.

Previous studies demonstrated that memory encoding and rapid generalization are dependent on the symmetrical interhemispheric dCA1 circuit (Zhou et al., 2017). Our results showed that the interhemispheric CA1 connection only occurred in dCA1 not vCA1 PNs.

Previous studies using classical tracing methods have established that CA2 forms functional synaptic connections with CA1 cells with a vast caudal spread along the longitudinal axis of the rat hippocampus (Tamamaki et al., 1988; Shinohara et al., 2012; Kohara et al., 2014; Dudek et al., 2016). However, the degree to which this longitudinal spread differs for dCA1 and vCA1 PNs is unclear. Our results showed that the connection strength of CA2 to dCA1 PNs was significantly stronger than CA2 to vCA1 PNs bilaterally. Different innervation strengths may suggest differential information processing efficiency in the dendrites of the dCA1 and vCA1 PNs.

Our evaluation revealed distribution details about the CA3–CA1 circuitry. The CA3–CA1 connectivity was organized as a topographic gradient. The input neurons in CA3 of dCA1 PNs exhibited a decreasing gradient bilaterally along the dorsoventral axis, while that of vCA1 PNs presented the increasing gradient on both hemispheres. Meanwhile, our findings demonstrated that the dCA1 PNs received much stronger overall CA3 inputs than vCA1 PNs. It could be speculated that the CA3-associated Schaffer collateral and contralateral commissural inputs have much stronger influence on the neural activity of dCA1 than vCA1 PNs.

Previous studies had confirmed that ENT directly projects to the hippocampus (Amaral and Witter, 1989; Witter et al., 2000; van Strien et al., 2009; Kitamura et al., 2015; Li et al., 2017; Chenani et al., 2019). Complementing this work, our results illustrated that ENT domains projected bilaterally to vCA1, but only ipsilaterally to dCA1 PNs. Our findings of the differences between ENT-dCA1 and ENT-vCA1 connectivity may help elucidate the different mechanisms underlying ENT-CA1 circuit-related behavior.

### Monosynaptic Extrahippocampal Inputs to Different Subarea of CA1 Projection Neurons

Previous researches have confirmed that CA1 is highly connected with brain regions beside HIP. We found some more extrahippocampal brain regions made monosynaptic projections to dCA1 or vCA1 PNs with differential distributions and connection strengths.

Previous studies employing traditional tracers have found some weak projections from vCA1 to ECT, but no feedback projection was mentioned. With the high-efficiency RV monosynaptic tracing system, we were able to notice that both dCA1 and vCA1 PNs received weak direct inputs from ECT. However, the ECT provided stronger connectivity to dCA1 than vCA1 PNs. Considering that ECT is an important isocortex subregion involved in sensory signals processing (Wang et al., 2012; Nishio et al., 2018), our results may provide new conjecture about how dCA1 PNs directly participate in information processing and memory formation in the cortex.

We also found that some brain regions only project to vCA1 PNs. For example, amygdala nuclei, RE, and PVT only provided direct input to vCA1 PNs. The amygdala nuclei are functionally associated with mental disorders including multiple anxiety disorders, addiction, and autism (Janak and Tye, 2015). Traditional tracing techniques have discovered that the BLA innervate CA1, along with limited inputs from other subregions of the amygdala nuclei (Yang et al., 2016; McDonald and Mott, 2017). Our results contributed more details about the projection preferences of different subregions in amygdala nuclei to vCA1 PNs. These results may provide more explanations as to how vCA1 PNs participate in amygdala-related functions.

Nucleus reuniens has been proven to modulate hippocampus-related navigation (Ito et al., 2018) and aversive memory consolidation (Troyner et al., 2018). Meanwhile, as a prominent component of the hypothalamo-pituitary-adrenal (HPA) axis, PVT has been reported to associate with stress-related anxiety and responsiveness (Wigger et al., 2004; Bergstrom, 2016; van Bodegom et al., 2017; Careaga et al., 2019). In previous studies with electrophysiological methods, the RE and PVT of the hypothalamus are reciprocally connected with both dCA1 and vCA1 (Zhang and Hernandez, 2013; Dolleman-van der Weel et al., 2017; Eichenbaum, 2017). Our data showed that only vCA1 PNs received direct inputs with RE and PVT. It may indicate that RE and PVT control dCA1 activity mostly through interneurons but not PNs.

Moreover, the previous CA1 lesion experiment confirmed that the vCA1, but not dCA1, is involved in sensory (odor) discrimination events (Roullet et al., 2005; Hunsaker et al., 2008; Kesner et al., 2010). Our tracing results may help to demonstrate that the anatomical evidence of subregions in OLFs made direct projections to vCA1 PNs in different connection strengths.

### Technical Considerations

Due to technical limitations, this study still has a few inadequacies that need to be improved. In order to get accurate results, we only target most dorsal or ventral areas which are not enough for whole dCA1 and vCA1 analysis. In recent studies of CA1 subdivisions, some single-cell RNA approaches such as multiplexed seqFISH mapping have suggested that the gene expression of CA1 cell types showed a gradual transition along the dorsoventral axis rather than having discrete boundaries between subregions (Malik et al., 2016; Shah et al., 2017; Soltesz and Losonczy, 2018; Cembrowski and Spruston, 2019). Therefore, without gene expression-specific classification of the dCA1 or vCA1 PNs, we can only analyze neuronal connectivity patterns of CA1 along the dorsoventral axis on the rather large scale of dorsal and ventral areas. In the experiments, we assumed that projection cells in the dorsal or ventral position shared the same input regions. In order to distinguish the different inputs of newly defined subpopulations of projection cells, additional criteria such as gene markers and comprehensive transcriptomes are needed for systematic examination and validation in subtype-specific connectivity of CA1 projection cells. Further studies using various mouse strains and new elaborate tracing systems are required to address the connectome of fine subdivisions in CA1.

## Conclusion

In summary, we mapped the input networks in the whole brain for PNs of two different subregions of CA1 by using a co-packaged rAAV helper virus and a modified RV tracing system, which allows us to target a limited subregion of CA1 with relatively high transfection efficiency. Our retrograde tracing experiments have determined statistically significant differences between whole-brain inputs of dCA1 and vCA1 PNs. Overall, our data showed that the dCA1 and vCA1 PNs shared similar monosynaptic upstream regions, but the comparative input strengths of the afferent connectivity may be related to distinct non-uniform information processing circuits. Our findings provide a necessary anatomical basis that may help elucidate the roles of dCA1 and vCA1 PNs in various behaviors, including the processing of parallel information.

## Materials and Methods

### Animals

All surgery and experimental procedures were performed in accordance with the guidelines of the Animal Experimentation Ethics Committee of Huazhong University of Science and Technology, and the Animal Care and Use Committees at the Wuhan Innovation Academy for Precision Measurement Science and Technology Chinese Academy of Sciences. Thy1-Cre mice (Jackson Laboratory stocknumber: 006143, a kind gift from Prof. Shumin Duan’s Lab of Zhejiang University) were mated with C57BL/6J mice which were purchased from Hunan SJA Laboratory Animal Company. All mice used in the experiments were young adult (8–12 weeks of age) male mice with weight of 20–25 g. C57BL/6 were used as control. Mice were group-housed in a quiet room with a 12/12-h light/dark cycle, and a thermostatically controlled environment with the temperature of 22–25°C. Food and water were provided *ad libitum*.

### Virus Preparation

We applied the RV-based monosynaptic tracing system and Thy1-Cre mice to identify the monosynaptic inputs of PNs in dCA1 and vCA1. All viruses used in this study were produced by our lab or provided by BrainVTA Science and Technology Company (Wuhan, China).

The retrograde trans-monosynaptic system consisted of the genetically modified EnvA-pseudotyped RV (RV-EnvA-ΔG-DsRed, 2.0 × 10^8^ infectious particles per ml) and the Cre-dependent co-packaged rAAV helper virus (AAV2/2, 5.27 × 10^12^ genomic copies per ml). Production of rAAVs and genetically modified RV were prepared as previously described (Sun et al., 2020). The co-packaged helper rAAV particles (AAV2/2) were constructed by cotransfection of two packaging plasmids AAV-EF1α-DIO-EGFP-TVA (GT) and AAV-EF1α-DIO-RVG into HEK293T cells at a ratio of 1:2. The RV, whose glycoprotein (RVG) gene was substituted to DsRed gene, was pseudotyped with an avian virus envelope protein (EnvA) (Figure 1A). The Cre-dependent rAAV helper virus encodes the GFP for the visualization of targeted neurons, TVA receptors for strict infection of RV, and the RVG protein for the transsynapptic spread of the modified RV as previously reported (Zhang et al., 2017). All viruses used in this study were stored at −80°C until use.

### Surgery Procedure

For trans-synaptic retrograde tracing, mice were anesthetized with sodium pentobarbital (80 mg/kg, i.p.) and fixed on stereotaxic apparatus (Item: 68030, RWD, Shenzhen, China). All viruses were injected with a pulled glass micropipette at a rate of 10 nl/min. The glass microelectrode remained for additional 10 min before withdrawal from the brain. The surface of mouse brain was embedded with lincomycin lidocaine gel before suturing. Mice were placed on electric heating blankets until entire recovery. After recovery, the mice were rehoused carefully, until being given additional injections or killed. The coordinates for the injection based on the Allen Mouse Brain Atlas were as follows: dCA1 (AP: −1.65 mm; ML: −1.20 mm; DV: −1.52 mm); vCA1 (AP: −3.10 mm; ML: −3.06 mm; DV: −4.00 mm). Sixty nanoliters of co-packaged rAAV helper (serotype = 2/2) was stereotactically injected into the dCA1 or vCA1, respectively (dCA1, *n* = 4; vCA1, *n* = 4).

Two weeks after rAAV helper virus injection into dCA1 or vCA1 of Thy1-Cre mice, we injected 120 nl of RV (RV-EnvA-ΔG-DsRed) in the same location. RV selectively infected TVA-expressing cells, which should be restricted to PNs in CA1 (Figure 1C). RV trans-synaptically spread to direct presynaptic cells after being complemented with RVG protein provided by rAAV. Mice were killed 7 days after RV injection for further analysis (Figure 1B).

We performed the same procedure in C57BL/6J mice as the control experiment to test the leakage of the tracing system. We found that a few RV-labeled DsRed-positive neurons were only detected at the injection site (Supplementary Figure 1). These results showed that our tracing system worked well with very little local leakage.

### Perfusion and Slicing

One week after RV injection, mice were transcardially perfused with 0.01 M PBS followed by 4% paraformaldehyde (PFA) in 0.01 M PBS. Brain tissue was carefully collected, postfixed overnight at 4°C, dehydrated in 30% sucrose in PBS for 48–72 h, and 50-μm frozen sections were performed across the whole brain with the freezing microtome (CryoStar NX50 cryostat, Thermo Fisher Scientific, San Jose, CA, United States). All continuous brain slices were collected one by one in a strict sequence in a 24-well plate with antifreeze liquid (50% = PBS, 20% = glycerine, and 30% = ethylene glycol) for further staining and imaging.

### Imaging

All brain slices were imaged with the virtual microscopy slide scanning system (VS120, Olympus, Japan) or confocal laser scanning system (SP8, Leica, Germany) for further analysis.

### Cell Counting and Data Analysis

To map the whole-brain monosynaptic distribution of the RV-labeled presynaptic neurons of CA1 PNs, we imaged serial coronal sections and analyzed them. For cell counting, we manually examined each brain slice to pinpoint the locations of the postsynaptic starter cells (coexpressing GFP and DsRed) (Figure 1C) and the labeled presynaptic neurons (expressing DsRed only). We marked each neuron according to their locations in anatomical brain region or subregion for specific region quantification according to the ARA2011 (Figure 2A). The numbers of all the marked cells were then counted and analyzed as reported (Do et al., 2016); meanwhile, their location details were classified and registered in the reference atlas. For quality control, we double checked the cell numbers in some subregions using the cell-counter plugin in ImageJ.

The Allen Mouse Brain Atlas (ARA2011) was used as the criterion to perform quantitative analysis of every brain section across different brain samples. In this study, the divisions of major brain regions and discrete subregions were mainly defined according to the Allen Brain Atlas 2011 (Figures 2B,C). Specifically, the whole brain was divided into eight major brain regions, comprising the PAL, STR, OLF, isocortex, HIP, CTXsp, TH, and HY. Each major brain region was further subdivided into several discrete brain subregions for better analysis of transverse or dorsoventral distribution. All related subregions and their abbreviations are listed in Table 1.

**TABLE 1:** Abbreviations.

**Abbreviation**	**Definition**
**PAL**	**Pallidum**
MS	Medial septal nucleus
NDB	Diagonal band nucleus
**STR**	**Striatum**
LSX	Lateral septal complex
sAMY	Striatum-like amygdalar nucleus
**OLF**	**Olfactory areas**
PIR	Piriform area
NLOT	Nucleus of the lateral olfactory tract
COA	Cortical amygdalar area
PAA	Piriform-amygdalar area
TR	Postpiriform transition area
**Isocortex**	
ECT	Ectorhinal area
**HIP**	**Hippocampal region**
CA1	Field CA1
CA2	Field CA2
CA3	Field CA3
ENT	Entorhinal area
ENTl	Entorhinal area lateral part
ENTm	Entorhinal area medial part
**CTXsp**	**Cortical subplate**
BMA	Basomedial amygdalar nucleus
BLA	Basolateral amygdalar nucleus
BLAa	Basolateral amygdalar nucleus anterior part
BLAp	Basolateral amygdalar nucleus posterior part
BLAv	Basolateral amygdalar nucleus ventral part
LA	Lateral amygdalar nucleus
**TH**	**Thalamus**
PVT	Paraventricular nucleus of the thalamus
RE	Nucleus of reuniens
VENT	Ventral group of the dorsal thalamus
ATN	Anterior group of the dorsal thalamus
MED	Medial group of the dorsal thalamus
ILM	Intralaminar nuclei of the dorsal thalamus
**HY**	**Hypothalamus**
LPO	Lateral preoptic area
LHA	Lateral hypothalamic area

*The bold terms and normal terms in Table 1 stand for brain regions and subregions, respectively.*

Then, the total number of the input neurons within the whole-brain or a certain brain region was quantified by adding up the numbers of the DsRed+ neurons within all involved brain areas. For precise quantitative evaluation of the whole-brain monosynaptic input distribution patterns in each tracing group, the number of the input neurons within a certain brain region/subregion/lamina was evaluated relative to the total number of the defined input neurons in the whole brain/a certain major brain region/subregion, and the proportions of whole-brain inputs/certain brain region inputs/a certain brain subregion were quantified and analyzed individually. The quantitative comparison of the distribution patterns of the input neurons between the two tracing groups was performed as the input connection strength index (CSI, defined as the ratio of the number of rabies-labeled presynaptic neurons versus the number of starter neurons).

For statistical comparisons, two-tailed unpaired Student’s *t*-tests were performed between two tracing groups. For statistical comparisons across more than two data, one-way ANOVA tests followed by Bonferroni tests were used for determining statistical differences using SPSS (version 22.0), with the significance set at ^∗^*P* < 0.05, ^∗∗^*P* < 0.01. All data values were presented as mean ± SEM.

## Data Availability Statement

The original contributions presented in the study are included in the article/Supplementary Material, further inquiries can be directed to the corresponding author/s.

## Ethics Statement

The animal study was reviewed and approved by the Animal Care and Use Committees at the Wuhan Innovation Academy for Precision Measurement Science and Technology Chinese Academy of Sciences.

## Author Contributions

ST, SJ, and FX designed the experiments. ST, SJ, YW, JP, and YZ performed the experiments. ST and SJ collected the data. ST analyzed the data and generated the figures. XH helped the virus preparation. ST and FX contributed to manuscript writing. XY, SJ, QL, and FX contributed to manuscript modification. All authors contributed to the article and approved the submitted version.

## Conflict of Interest

The authors declare that the research was conducted in the absence of any commercial or financial relationships that could be construed as a potential conflict of interest.
