# Supplementary material for: Whole-Brain Mapping the Direct Inputs of Dorsal and Ventral CA1 Projection Neurons
Source: Front Neural Circuits. 2021 Apr 14;15:643230. doi: 10.3389/fncir.2021.643230 (PMC8079783; doi:10.3389/fncir.2021.643230)
Supplement: Supplementary file 1 [file Data_Sheet_1.DOCX]

Supplementary Material

# Supplementary Table 1：Quantifications of starter cells, total input cells and convergence index of dCA1 and vCA1 groups for Figure 1.

| Total number of starter cells | | | | | |
| --- | --- | --- | --- | --- | --- |
|  | 1#mouse | 2#mouse | 3#mouse | 4#mouse | Mean±SEM |
| dCA1 | 768 | 847 | 719 | 750 |  |
| vCA1 | 1950 | 1805 | 2009 | 1225 |  |
|  |  |  |  |  |  |
| Total number of input cells across the whole brain | | | | | |
| dCA1 | 21526 | 23520 | 24727 | 23990 |  |
| vCA1 | 50662 | 48524 | 58357 | 37971 |  |
|  |  |  |  |  |  |
| Convergence Index | | | | | |
| dCA1 | 28.03 | 27.77 | 34.39 | 31.99 | 30.54±1.60 |
| vCA1 | 25.98 | 26.88 | 29.05 | 31.00 | 28.40±1.15 |

# Supplementary Table 2：Quantifications of Distributions of ipsilateral and contralateral input neurons within 5 subregions of HPF for Figure 5.

| Groups | Subregions | Data value |
| --- | --- | --- |
| dCA1 tracing group | CA2 | 69.80%±1.72% for ipsi vs contra 30.20%±1.72% |
|  | CA3 | 55.67%±1.27% for ipsi vs contra 44.33%±1.27% |
|  | ENT | 100% for ipsi vs contra 0% |
|  |  |  |
| vCA1 tracing group | CA2 | 96.99%±0.78% for ipsi vs contra 3.01%±0.78% |
|  | CA3 | 70.04%±0.64% for ipsi vs contra 29.96%±0.64% |
|  | ENT | 91.68%±1.94% for ipsi vs contra 8.32%±1.94% |

# Supplementary Figure 1：


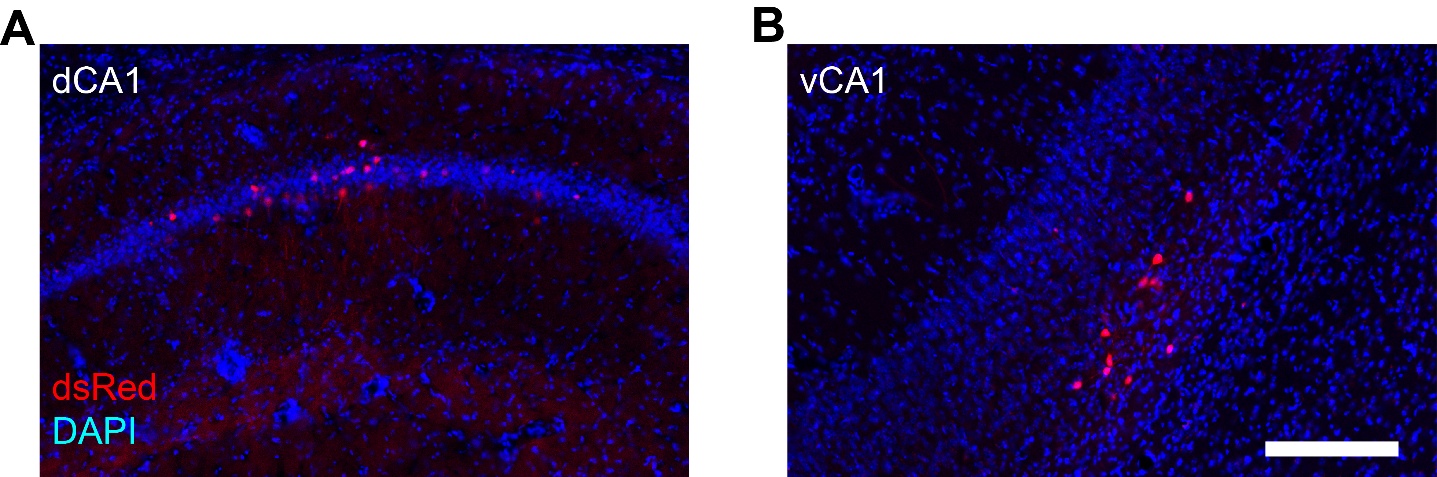


**Supplementary Figure 2.** Representative images of coronal brain sections after injecting rAAV helper virus and RV-EnvA-ΔG-dsRed into wild-type mice. (A) Coronal brain section containing the injection site (dorsal CA1) showed a very limited number of EnvA-dsRed positive neurons. (B) Coronal brain sections containing the ventral CA1 showed few RV labeled neurons. Scale bar: 200 µm.
